# Hyposmia Is Associated with RBD for PD Patients with Variants of *SNCA*

**DOI:** 10.3389/fnagi.2017.00303

**Published:** 2017-09-20

**Authors:** Yuanyuan Li, Wenyan Kang, Linyuan Zhang, Liche Zhou, Mengyue Niu, Jun Liu

**Affiliations:** Department of Neurology, Institute of Neurology, Ruijin Hospital Affiliated to School of Medicine, Shanghai Jiaotong University Shanghai, China

**Keywords:** Parkinson's disease, RBD, hyposmia, autonomic symptoms, single nucleotide polymorphisms

## Abstract

**Objective:** Hyposmia may occur simultaneously with REM sleep behavior disorder (RBD) as a specific phenotype in Parkinson's Diseases (PD), of which the disease progression is fast. In the study, we tried to identify whether the genotypic characteristics could participate in the co-occurrence of hyposmia and RBD in PD patients.

**Methods:** 152 PD patients were recruited from the Department of Neurology, Ruijin Hospital affiliated to Shanghai JiaoTong University School of Medicine from 2011 to 2016, with comprehensive clinical assessment performing. Two SNPs of *SNCA* (rs11931074 and rs894278) in 105 patients were also analyzed.

**Results:** Overall, 84 of 152 PD patients (55.3%) were diagnosed with RBD after PSG evaluation. After regression analysis, higher levels of three parts of UPDRS and SCOPA-AUT scores were all associated with increased risk of RBD in PD patients, respectively. While for olfactory function, we didn't find significant correlation between hyposmia and RBD in PD patients. However, we found that in the group of minor G allele of rs894278, patients with lower score of SS-16 had a 4.76-fold risk of suffering from RBD in patients (95% CI: 1.39–16.67; *p* = 0.013). Furthermore, we analyzed SNP associated gene expression by eQTL analysis in Genevar database and found that GG genotype of rs894278 was associated with higher levels of α-synuclein in Nerve tissue (*p* = 1.5E-8) while TT genotype of rs11931074 was associated with higher levels of α-synuclein in Brain (*p* = 0.0082), which suggesting a potential functional relevance with different symptoms of PD.

**Conclusions:** Hyposmia was associated with RBD in PD patients with the minor G allele of rs894278, which represent one specific subtype of PD. This study could provide more detail information about PD subtype of RBD with hyposmia in the future.

## Introduction

Parkinson's disease (PD) is one of the most popular synucleinopathies characterized by core motor features, which often associated with non-motor manifestations, including sleeping disorders, olfactory loss, osteoporosis, personality changes, cognitive decline and autonomic abnormalities (Chaudhuri et al., 2006; Berg et al., 2015; Gao et al., 2015; Wei et al., 2016). Rapid eye movement sleep behavior disorder (RBD) is one of them, which is characterized by the loss of normal skeletal muscle atonia during REM sleep, accompanied with prominent motor activity in dream event (Boeve et al., 2007). Currently, one of the known strong identifiers of prognosis subtype is the co-occurrence of RBD and PD (Arnaldi et al., 2016). Previous study has been shown that RBD was associated with longer PD duration and higher dosage of dopaminergic therapy (LED), suggesting that the PD with RBD subtype may also have a different risk factor profile (Lee et al., 2010). Hyposmia is another common non-motor symptom, and 70–90% PD patients suffer from olfactory impairment according to previous studies (Double et al., 2003).

Recently, It has been reported that olfactory dysfunction and RBD may occur simultaneously in some PD patients as a specific subtype of disease, in which patients were more likely to have akinetic-rigidity and cognitive dysfunction (Kang et al., 2016). However, the association between olfactory dysfunction and RBD in PD patients was not consistent (Jankovic et al., 2015; Arnaldi et al., 2016; Wan et al., 2016), at the same time, the underlying mechanism in RBD being co-occurrence with and hyposmia of PD patients was still not clear.

Previous studies found that carriers of *Parkin* and *LRRK2* mutation more likely suffered from olfactory dysfunction (Khan et al., 2004, 2005), which is meaning that genotypic characteristics could affect the association between RBD and hyposmia in PD patients. We have demonstrated that hyposmia was correlates with *SNCA* variant in PD patients (Chen et al., 2015), so we performed the study to investigate whether the two typical SNPs of *SNCA* variants contribute to the co-occurrence hyposmia and RBD in PD.

## Methods

### Participants

All study participants were recruited from the Department of Neurology, Ruijin Hospital affiliated to Shanghai JiaoTong University School of Medicine from 2011 to 2016. 152 patients with Parkinson Disease (PD) were enrolled in our study with the diagnosis made according to the UK Brain Bank or current clinical diagnostic criteria by at least two movement disorders specialists (Hughes et al., 1992). Ages of the all participants were ranging from 50 to 80 years old, and patients with psychiatric disease or severe dementia were excluded. This study was approved by the ethics committee of Ruijin Hospital, Shanghai JiaoTong University School of Medicine, Shanghai, China, and all participants or their guardians provided written informed consent.

### Demographics and clinical assessment

For these 152 PD patients, the disease stage was determined using the modified Hoehn and Yahr (HY) staging (range 0–5) (Hoehn and Yahr, 1967). The motor subscale of the Unified Parkinson's Disease Rating Scale (UPDRS II-III) was used to evaluate motor symptoms. Moreover, a series of comprehensive questionnaires besides the evaluating of motor symptoms were also administered for every patient, including the non-motor symptom quest (NMSQ), REM sleep behavior disorder screening questionnaire (RBDSQ), scale for outcomes in PD-Autonomic (SCOPA-AUT), olfaction test (SS-16), Hamilton Depression Rating Scale-17 (HAMD-17) and Mini-Mental State Examination (MMSE). NMSQ was used to evaluate non-motor symptoms and SCOPA-AUT to assess autonomic dysfunction (Verbaan et al., 2007). An olfaction test (SS-16) was also performed to assess the olfactory function through 16-item odor identification (Hummel et al., 1997; Chen et al., 2012). HAMD-17 was administered to measure depressive symptoms (Hamilton, 1960), and MMSE was performed to evaluate the cognitive function resulting from PD (Zhang et al., 1990). Receiver operating characteristic (ROC) curves were plotted by calculating sensitivity and 1-specificity for specific cut-off values. Of the 152 PD patients, 84 were affected by RBD meeting the International Classification of Sleep Disorder (ISCD)-II criteria (American Academy of Sleep Medicine, 2005) and overnight video-polysomnograph (PSG) examination was done for each patient to confirm the diagnosis.

### Selection of SNPs and genotyping

Our previous studies were shown that *SNCA* rs894278 and rs11931074 were distributed in most of Chinese PD patients (Chen et al., 2015). So we measured the both SNPs in the recruited patients. Genomic DNA for 105 of the 152 PD patients was isolated from lymphocytes of whole blood using the QIAamp DNA extraction kit (Qiagen, Valencia, CA) and genetic information of was obtained by direct DNA sequencing performed on a 3730xl DNA analyzer (Applied Biosystems, Foster City, CA, USA) as we reported (Liu et al., 2013).

### Analysis of expression quantitative trait loci (eQTL), functional characterization and mutation frequency of the two SNPs

The potential functional impact of validated SNPs on gene expression was evaluated by analyzing gene-SNP association in expression quantitative trait loci (eQTL) studies with the Genevar database available in the HapMap3 dataset (Yang et al., 2010). While the predicted regulatory motif function of each SNP was predicted using ENCODE project explored from UCSC databased (http://www.broadinstitute.org/mammals/haploreg/haploreg.php).

### Statistical analysis

Data were analyzed by the SPSS software version 20.0 (SPSS Inc., Chicago, IL, USA). Statistical significance was taken as two-sided *p* < 0.05. Categorical variables were compared using the χ^2^ or Fisher exact test to assess the difference of baseline parameters between the PD with or without RBD group. Binary logistic regression analysis was used to determine the independent risk factors of RBD in PD patients and effect-size estimates were expressed as odds ratios (ORs) and 95% confidence intervals (CIs). For statistical analysis between the subgroups, chi-square tests were used for categorical variables and students' tests for continuous variables. SNP analysis was performed by binary logistic regression analysis adjusting for age, sex, smoking and disease duration. The multivariable analysis was repeated for the replication patients.

## Results

### Demographic and clinical features

The main clinical and demographic features of patients were listed in Table 1. Eighty four of 152 PD patients were affected by RBD (PD+RBD, 55.3%). For these patients, gender proportion was difference: 79.8% were man in PD+RBD group compared to 52.9% in PD without RBD group (*p* < 0.001), and patients with RBD presented longer disease duration and higher UPDRS (I-III) scores than PD without RBD group (*p* = 0.046 for PD duration; *p* < 0.001 for UPDRS I; *p* = 0.002 for UPDRS II; *p* = 0.017 for UPDRS III). Moreover, there were more smokers (*p* = 0.041) and more patients suffering constipation in patients with RBD (*p* = 0.002, Table 1).

**Table 1:** Demographic and clinical features in PD patients with or without RBD.

	**With RBD (*n* = 84)**	**Without RBD (*n* = 68)**	***P***
Age (years)	64.2 ± 7.4	62.8 ± 7.9	0.267
Sex			
Female	17 (20.2%)	32 (47.1%)	<0.001
Male	67 (79.8%)	36 (52.9%)	
Smoking			
Yes	21 (26.3%)	8 (12.5%)	0.041
No	59 (73.8%)	56 (87.5%)	
Constipation			
Yes	41 (64.1%)	22 (36.7%)	0.002
No	23 (35.9%)	38 (63.3%)	
PD duration (years)	4.7 ± 4.1	3.5 ± 3.4	0.046
HY	1.9 ± 0.8	1.7 ± 0.8	0.096
UPDRS-I	3.4 ± 2.2	1.9 ± 1.7	<0.001
UPDRS-II	10.7 ± 4.3	8.1 ± 4.3	0.002
UPDRS-III	19.4 ± 10.4	15.4 ± 9.5	0.017

*PD, Parkinson's disease; RBD, REM sleep behavior disorder*.

### Analysis of clinical characteristics and SNPs between PD with or without RBD

Due to the importance of RBD in PD disease, we further evaluated the clinical manifestation between PD with or without RBD. After logistic analysis, as shown in Table 2, patients with higher NMSQ scores were shown 11.7-fold (95% CI: 4.4–31.5; *p* < 0.001) higher risk in PD+RBD group. In terms of autonomic dysfunction, the odds of having worse syndromes in PD+RBD patients were 6.1 times (95% CI: 2.6–14.5; *p* < 0.001) risk. While for the influence of genetic characteristics, Table 3 was listed genotype distributions of rs894278, rs11931074 variants in the patients with and without RBD. After logistic regression analysis, adjusted by age, sex, smoking and PD duration, we didn't find significant different between gene variations and RBD occurrence in PD patients of dominant, recessive or additive model.

**Table 2:** Evaluation of the PD patients with or without RBD.

	**With RBD (*n* = 84)**	**Without RBD (*n* = 68)**	**OR (95% CI, adjusted)**	***P***
**NMSQ**				
< 8	27 (32.1%)	50 (84.7%)	1	<0.001
≥8	57 (67.9%)	9 (15.3%)	11.7 (4.4–31.5)	
**SCOPA-AUT**				
< 9	13 (15.5%)	38 (57.6%)	1	<0.001
≥9	71 (84.5%)	28 (42.4%)	6.1 (2.6–14.5)	
**SS-16**				
< 8	48 (58.5%)	30 (46.2%)	1	0.566
≥8	34 (41.5%)	35 (53.8%)	0.8 (0.4–1.8)	
**HAMD-17**				
< 8	42 (50.6%)	38 (56.7%)	1	0.97
≥8	41 (49.4%)	29 (43.3%)	1.0 (0.3–3.4)	
**MMSE**				
< 26	14 (17.3%)	11 (16.4%)	1	0.827
≥26	67 (82.7%)	56 (83.6%)	0.9 (0.4–2.3)	
**LED**				
< 512.5	44 (73.3%)	36 (87.8%)	1	0.532
≥512.5	16 (26.7%)	5 (12.2%)	0.7 (0.2–2.2)	

*PD, Parkinson's disease; NMSQ, non-motor symptom quest; SCOPA-AUT, Scale for outcomes in PD-Autonomic; SS-16Q, Sniffin' Sticks 16 types tests; RBD, REM sleep behavior disorder; HAMD-17, Hamilton Depression Rating Scale-17; MMSE, Mini-Mental State Examination; LED, L-dopa Equivalent Dosage. All results were adjusted for age, sex, PD duration, constipation and smoking. OR, odd ratio; 95% CI, 95% confidence interval*.

**Table 3:** Association of SNPs in PD patients with or without RBD.

**SNP**	**Gene**	**Minor allele**	**Dominant**		**Recessive**		**Additive**	
			**OR (95% CI)**	***P***	**OR (95% CI)**	***P***	**OR (95% CI)**	***P***
rs894278	*SNCA*	G	0.87 (0.28–2.65)	0.801	0.81 (0.33–1.96)	0.633	0.94 (0.52–1.73)	0.850
rs11931074	*SNCA*	T	2.19 (0.62–7.69)	0.224	0.85 (0.36–2.05)	0.724	0.76 (0.41–1.41)	0.379

*All results were adjusted for age, sex, PD duration and smoking. OR, odd ratio; 95% CI, 95% confidence interval*.

### Correlation between hyposmia and RBD in PD patients classified by gene variants

To verify whether genotypic characteristics would participate in the phenotype of hyposmia, we further evaluated the correlation between hyposmia and RBD in PD patients with different variants of rs894278 and rs11931074. The result indicated that PD patients with lower score of SS-16 had a 4.76-fold risk of suffering from RBD in the minor G allele of rs894278 (95% CI: 1.39–16.67; *p* = 0.013, Table 4). For the patients with the minor G allele of rs894278, 57.6% patients with SS-16 score below 8 exhibit RBD while only 23.1% didn't. As for the minor T allele of rs11931074, patients with decreased olfactory score were more incline to manifest RBD, but didn't achieve significant difference (*p* = 0.082, Table 4). 43.5% PD patients with SS-16 score below 8 in the T allele of rs11931074 exhibit RBD while 16.7% not.

**Table 4:** Analysis of olfactory function in patients with or without gene variants of *SNCA*.

	**With RBD**	**Without RBD**	**OR (95% CI)**	***P***		**With RBD**	**Without RBD**	**OR (95% CI)**	***P***
**rs894278**									
GG+GT					TT				
≥8	14 (42.4%)	20 (76.9%)	1	0.013		13 (50.0%)	12 (75.0%)	1	0.435
<8	19 (57.6%)	6 (23.1%)	4.76 (1.39–16.67)			13 (50.0%)	4 (25.0%)	1.82 (0.40–8.33)	
**rs11931074**									
TT+GT					GG				
≥8	13 (56.5%)	15 (83.3%)	1	0.082		15 (41.7%)	17 (70.8%)	1	0.289
<8	10 (43.5%)	3 (16.7%)	4.17 (0.83–20.0)			21 (58.3%)	7 (29.2%)	1.96 (0.56–6.67)	

*RBD, REM sleep behavior disorder. All results were adjusted for age, sex, PD duration and smoking. OR, odd ratio; 95% CI, 95% confidence interval*.

### Functional prediction of rs894278 and rs11931074

**Figure 1:**
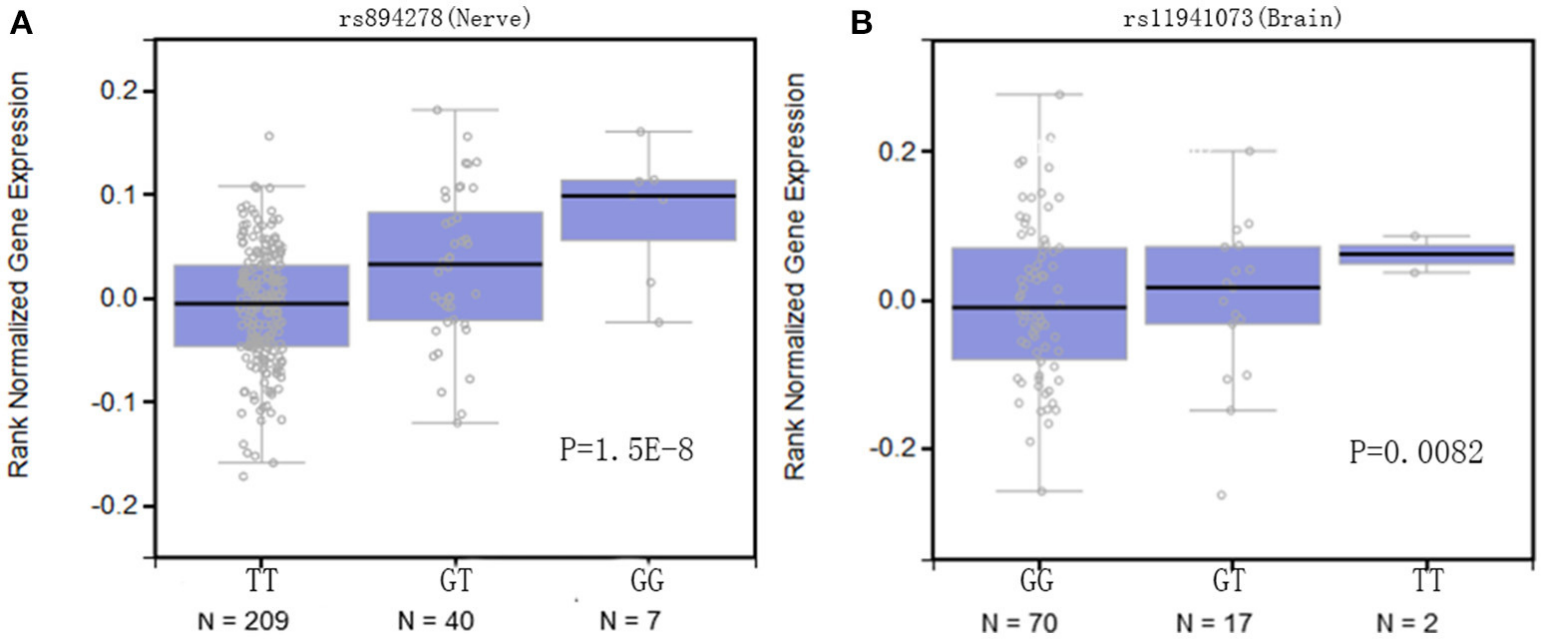
Boxplots of *SNCA* expression from Genevar data. Below each boxplot is the number of individuals with each genotype. **(A)** GG carriers of rs894278 was associated with higher levels of *SNCA* in the Nerve (*p* = 1.5E-8); **(B)** TT genotype of rs11931074 was associated with higher levels of *SNCA* in the Brain (*p* = 0.0082).

To fully understand the functional impacts of the identified SNPs of *SNCA* related to hyposmia and RBD, the SNP associated gene expression was tested by eQTL analysis in Genevar database (Figure 1). The results showed both SNPs were significantly associated with the gene expression in normal tissues: GG genotype of rs894278 was associated with higher levels of *SNCA* in Nerve (*p* = 1.5E-8) while TT genotype of rs11931074 was associated with higher levels of *SNCA* in Brain (*p* = 0.0082). On the other hand, the potential impact of the two SNPs on gene function was analyzed using HaploReg. Based on bioinformatics analysis from the ENCODE project, the two SNPs were suggested to potentially affect transcription factor binding and have potential regulatory roles (Table 5), which rs894278 may be located in a predicted GCNF, Nanog_disc4, Nkx2_9, TATA_disc9, and Zfp410 motifs, and rs11931074 may be located in predicted SETDB1_disc1 motif. The above suggest that two SNPs of *SNCA* have potential functional relevance with PD.

**Table 5:** Functional prediction of SNPs using data from ENCODE.

**Variables**	Motifs^*^	**Strand**	**Ref**	**Alt**
rs894278	GCNF	−	−7	4.9
	Nanog_disc4	+	10.6	12.9
	Nkx2_9	+	11.9	14.7
	TATA_disc9	−	0.8	12.8
	Zfp410	−	11.4	10.4
rs11931074	SETDB1_disc1	+	9	8.4

ENCODE, the Encyclopedia of DNA Elements;

* *Evidence of alteration in regulatory motif*.

## Discussion

To the best of our knowledge, the present study is the first to investigate the influence of genotypic characteristics on the association between RBD and hyposmia in PD patients. The most noteworthy finding was the identification of one promising SNP, *SNCA* rs894278, in which the patients harboring minor G allele was more likely to have hyposmia and RBD simultaneously, and may have worse disease progression.

Numerous studies have investigated many factors associated with the presence of RBD in PD patients. In our study, we found that PD+RBD patients had male predominance and longer disease duration, which was in line with previous studies (Gjerstad et al., 2008; Yoritaka et al., 2009; Poryazova et al., 2013). Moreover, PD+RBD patients were presented with higher UPDRS (II–III) scores, which was consistent with previous study of factors related to RBD in 2010 (Lee et al., 2010) and study in 2014 (Gong et al., 2014). Furthermore, after risk regression analysis of developing RBD, several non-motor symptoms appeared increasing incidence. Previous studies have shown autonomic dysfunction including autonomic symptom scores assessing, formal autonomic testing and cardiac MIBG scintigraphy have been related to idiopathic RBD, as also found in this study (Miyamoto et al., 2006; Postuma et al., 2008; Yoritaka et al., 2009). As for olfactory function, hyposmia didn't appear to be a risk factor for RBD of PD patients in our study, which was in line with one previous study of 2015 (Jankovic et al., 2015). However, there are also studies showed that hyposmia may simultaneously occurred with RBD as a specific subtype of PD, in which patients were more likely to suffer from cognitive dysfunction and exhibit akinetic-rigid-type (Kang et al., 2016).

Previous studies have shown that PD have different genetic heterogeneity and phenotypic imprecision. A-synuclein (*SNCA*) is a hallmark of PD and crucial factor in PD pathogenesis. Clinical-pathological studies have also suggested that both hyposmia and RBD may be related to a-synuclein deposition (Ubeda-Banon et al., 2010). On the other hand, we have demonstrated that hyposmia was correlates with *SNCA* variants in PD patients in our previous study (Chen et al., 2015), thus it is worth exploring whether these variants are associated with the specific subtype in PD. Rs894278 and rs11931074 SNPs in *SNCA* has been identified as conferring the strongest PD risk (Liu et al., 2013) and recently, studies have shown these two SNPs were also associated with some other phenotypes in PD, such as festination (Zheng et al., 2017) and earlier onset of PD (Huang et al., 2015). So, we further evaluated these two SNPs to identify the relationship, and the result indicated that hyposmia were associated with RBD in PD patients with minor G allele of rs894278, which means PD patients harboring this allele was more likely to have hyposmia and RBD simultaneously and worse disease progression.

On the other hand, functional characterization might be important to dissect the mechanistic basis for the variants' association with clinical subtypes, thus we explored the gene expression by eQTL analysis in Genevar public database. And the result was shown that the genotypes of two SNPs were significantly correlated with expression of α-synuclein in tissues or cell lines, which suggested a potential functional relevance with PD. The study with clinical and genetic information would help us to explore the progression and precision management for a subtype of RBD with olfactory dysfunction in PD patients. Further studies on the mechanism of how these variants influenced the pathogenesis and progression of PD are needed in the future study.

In conclusion, we found that PD with RBD patients had higher scores in UPDRS three parts, and more NMS and autonomic dysfunctions, especially constipation. Moreover, patients with hyposmia were co-occurrence with RBD in the minor G allele of rs894278, which would present one specific subtype of PD.

## Ethics statement

This study was carried out in accordance with the recommendations of the “ethics committee of Ruijin Hospital, Shanghai JiaoTong University School of Medicine, Shanghai, China” with written informed consent from all subjects. All subjects gave written informed consent in accordance with the Declaration of Helsinki. The protocol was approved by the “ethics committee of Ruijin Hospital, Shanghai JiaoTong University School of Medicine, Shanghai, China.”

## Author contributions

JL conceived, supervised the project and contributed to patients' recruitment. YL, JL, and WK drafted the manuscript. YL, WK, LZha, LZho, and MN contributed to sample collection, performed data management and statistical analyses. All authors read and approved the final version of the manuscript.

### Conflict of interest statement

The authors declare that the research was conducted in the absence of any commercial or financial relationships that could be construed as a potential conflict of interest.

## Abbreviations

PD: Parkinson's disease
iRBD: idiopathic rapid-eye-movement sleep behavior disorder
PSG: polysomnograph
SNP: single nucleotide polymorphisms
NMSQ: non-motor symptom quest
SCOPA-AUT: scale for outcomes in PD-Autonomic
HAMD-17: Hamilton Depression Rating Scale-17
MMSE: mini-mental state examination
eQTL: expression quantitative trait loci
OR: odds ratio.
